# Telepractice as a Reaction to the COVID-19 Crisis: Insights from Croatian SLP Settings

**DOI:** 10.5195/ijt.2020.6325

**Published:** 2020-12-08

**Authors:** Jelena Kuvač Kraljević, Ana Matić, Katarina Pavičić Dokoza

**Affiliations:** 1 Department of Speech and Language Pathology, University of Zagreb, Croatia; 2 Polyclinic for the Rehabilitation of Listening and Speech (SUVAG), Croatia

**Keywords:** Client selection, COVID-19, Direct therapy, SLP satisfaction, Telepractice

## Abstract

Telepractice facilitates services in exceptional settings and situations. The ongoing COVID-19 pandemic is certainly such a situation. Due to pandemic-related restrictions, speech-language pathologists (SLPs) needed to adopt new approaches to their professional functioning. The aim of the paper is to examine SLP professionals' perceptions and application of telepractice in SLP settings in Croatia during the COVID-19 pandemic. Two hundred and fifty-five SLPs completed an online survey. The results demonstrated that most SLPs had provided direct online therapy, mainly those employed in health care and private practice. The chief reasons for clients' refusal of therapy delivered via telepractice included the lack of equipment, insufficient independence, and doubts on the effectiveness of telepractice. Although only 3% of SLPs had acquired some formal knowledge of telepractice before the pandemic, over 70% expressed satisfaction with telepractice because it allowed them to provide undisturbed clinical services in an exceptional situation.

According to the World Health Organization (2016), telehealth is defined as the delivery of health care services using information and communications technology (ICT). Telehealth can be used for diagnosis and treatment of diseases and injuries, research and evaluation, for continuing education of health professionals, and for proper exchange of information; synchronous (i.e., interactive services in real time); and asynchronous (i.e., material or data storage and their use or analyses apart from any real time intervention). It can contribute to the achievement of universal health coverage by improving access to services wherever those in need of them may be. This especially refers to the population residing in remote areas, vulnerable groups, and ageing populations.

The term telepractice can be used interchangeably with telehealth and will be used throughout this article to describe speech and language services delivered through a telehealth service delivery model. Key considerations when using telepractice for speech and language pathology (SLP) include accessibility, environments, regulation, legislation (i.e., reimbursement or insurance coverage), tele-ethics, competency, and preparation for service delivery (Cason & Cohn, 2014).

Environments include different situational settings (e.g., client's home, SLP's office) when using telepractice. Legislation policies, licensure and reimbursement coverage are country-specific and sometimes dependent on the system of employment. This is the case in Croatia, where codes of practice within different systems define the scope of SLP activities (Kuvač Kraljević et al., 2019). Due to great variability of state laws and regulations across countries and systems, professionals are individually obliged to abide by all state requirements, which have recently been updated according to the ongoing COVID-19 pandemic (see ASHA, 2020).

Tele-ethics is a concept which encompasses all components covered by ethics in general, with some additional specific issues. In a broad sense it refers to one's professional responsibility to uphold the client's well-being. In a more specific sense, tele-ethics includes a professional's duty of care towards the client (i.e., SLPs' knowledge of techniques, technologies and research); equivalence of services (i.e., the provided service and its results must not differ in in-person and remote settings); privacy of information and place (i.e., all information collected via online tools and platforms should remain private and, if stored, be adequately protected); and the equity of access (i.e., telepractice should enable access to service, and must not be misused for any form of discrimination). Examples of best ethical practices for telepractice are outlined in Cohn (2012).

Preparation for telepractice includes thorough consideration of the general needs for services, organisational and environmental assessment, practitioner readiness (i.e., self-perceived confidence and practice competence), selection of optimal technologies and tools, as well as client selection (i.e., which client might benefit from this service delivery model and why).

This paper will mainly discuss the latter (i.e., the preparation for the delivery of telepractice applied to the current COVID-19 situation and the crisis-induced change).

## APPLICATION AND EFFECTIVENESS OF TELEPRACTICE: CURRENT FINDINGS

Telepractice within speech and language therapy is still relatively new, which is why the efficacy research is limited, especially meta-analysis studies. A 2020 analysis of the PubMed database revealed that less than 1% of all papers on telepractice were published during the 1990s, and approximately 82% were published between 2010-2020. This significant rise in the number of published papers during the last 30 years is proof of changing practice trends, and the increasing importance of telepractice in recent years.

Nonetheless, the majority of available studies lack detailed description of the preparation aspects, such as the issue of *setting* (with respect to the client's home and the professional's office environment), *professional readiness* (level of education and feelings of competence), and *client selection* (as the driving-force for adequate service to those who are most likely to benefit). These factors may impact the provision and effectiveness of telepractice (Weidner & Lowman, 2020).

Studies with younger populations with diverse impairments are generally lacking. In addition, there are limited high quality study designs such as randomized control trials (RCTs) with a matched control group (ASHA, 2019; Weidner & Lowman, 2020). These types of studies would enhance the efficacy research and be more generalisable and applicable to (tele)clinical settings.

A small amount of available data usually reports effectiveness and usefulness of telepractice in the adult population, mostly patients with post-stroke aphasia or Parkinson's disease (Hall, et al., 2013), communication and swallowing disorders (Molini-Avejonas, et al., 2015) or stuttering (McGill et al., 2019). Parsons et al. (2017) reported a gain in parents' knowledge of treatment strategies which resulted in increased fidelity of indirect therapy delivered to their children with autism spectrum disorder (ASD). There are certain findings on the positive impact of telepractice on service providers, as well. Finch et al. (2020) observed increased confidence of less experienced SLPs when communicating with the severely impaired population of adults with aphasia.

Those who perceive this relatively novel service delivery model for providing speech and language services point out several beneficial characteristics. It provides SLPs and their clients with the opportunity to work towards achieving clients' goals outside of strictly clinical settings, thus easing and speeding up the process of transferring to natural settings. Furthermore, it aids in overcoming distance and travel issues and the expenses that usually accompany them. Finally, it helps fill service gaps in educational and adult health care settings (ASHA, 2019; Cason & Cohn, 2014; Weidner & Lowman, 2020). Despite the obvious advantages of telepractice, SLPs must base their professional and client-related judgements on evidence. Special emphasis should be put on preparational aspects related to telepractice:

- Is the professional adequately educated and competent to use telepractice for the delivery of speech and language services?
- Who is telepractice intended for and under which circumstances should it be implemented?
- How can professionals ensure a proper setting for themselves and their clients?
- Which tools and equipment should be used?
- What are regulation requirements and ethical responsibilities?

Telepractice has not often been the interest of research in Croatia, and its use in clinical settings is still sporadic. Almost every SLP in Croatia uses various hardware and software (e.g., a specialized digital SLP set and different mobile applications developed mainly within the project ICT-AAC [ICT Competence Network for Innovative Services for Persons with Complex Communication Needs], (Ivšac Pavliša et al. 2012; Ivšac Pavliša et al. 2016) in the rehabilitation of various disorders. However, these are mostly used in *face-to-face* in-person therapy. There are only two papers focused on SLP telepractice in Croatia, and both are written from a technological, and not a therapeutic point of view.

Plantak Vukovac et al. (2015) administered a questionnaire that showed 68% of SLPs would be willing to provide telepractice, but mostly by asynchronous communication (i.e., sharing files). These professionals viewed telepractice as a valid tool for monitoring clients once the clinical treatment is over. Approximately 80% of SLPs use a computer in their daily work-related activities, whereas tablets, smartphones, and web and digital cameras are used to a lesser extent. In her second paper, Plantak Vukovac (2016) demonstrated video telepractice for children with articulation disorders designed in accordance with several principles that reduce cognitive load. The quality of the video was evaluated by the parents of children included in online therapy. They reported several disadvantages: low video quality as one of least favourable aspects of video in general; the level of their engagement required during online therapy; and loss of their children's attention. Interestingly, it was the clients who identified synchronous communication (i.e., real-time interactions) as an optimal mode of telepractice.

### TELEPRACTICE AS A REACTION TO GLOBAL COVID-19 PANDEMIC

Telepractice facilitates services in exceptional settings and situations. Many positive aspects of telepractice make this approach suitable in the case of any sort of natural disaster, as it allows for undisrupted clinical services (Cason & Cohn, 2014). Following this logic, the use of telepractice has increased during the global COVID-19 pandemic. Due to crisis-related working, moving, and travelling restrictions, most SLPs were forced to adopt new approaches to their professional functioning. Their daily working routine changed for SLPs who had formerly conducted services from their offices – either directly, with full responsibility for delivering the training, or indirectly, by supervising a family member (Boyle, 2007). The conduct of therapy away from their offices and online, using PCs, web-based technologies, tools and platforms was previously considered more an exception than a rule, but suddenly became the only accessible working arrangement.

The Comité Permanent de Liaison des Orthophonistes-logopèdes de l'ue, (CPLOL) is the Standing liaison Committee of EU speech and language therapists and Logopedists. The CPLOL made recommendations for SLPs throughout Europe to carry out telepractice whenever appropriate so that clients might continue to benefit from therapy despite the pandemic (CPLOL COVID-19 statement, 2020). Nevertheless, a recently conducted COVID-19 survey revealed that government authorities did not recommend telepractice in almost 40% of 27 European countries involved in the study. As for other countries, 48% reported that there was no mention of telepractice in legislation, and two countries reported that conducting telepractice was considered illegal (CPLOL Survey Report, 2020). Croatia falls under the 48% of countries which have not yet regulated telepractice.

The use of telepractice in response to the COVID-19 pandemic was more or less imposed, and the transition occurred extremely rapidly. The exact details of its implementation across various settings and amongst SLPs with different professional expertise; their years of clinical experience; and their work with clients of different ages, diagnoses, and social backgrounds, is not entirely clear. For most SLPs the implementation of completely new methods and tools for service provision occurred in the context of scarce knowledge of preferable settings and factors that should guide telepractice. Attaining a clearer insight into the present use of telepractice during the COVID-19-pandemic in largely understudied telepractice settings should be the first step toward informing more in-depth future analyses.

### PRESENT STUDY

The main aim of the current paper was to examine professionals' perceptions and their application of telepractice in SLP settings in Croatia during the COVID-19 pandemic. The specific aims were to analyse what drives professionals' and clients' choices in decision-making and to explore SLPs' attitudes and level of confidence towards adopting this novel approach to service delivery.

Several questions were formulated to address these goals:

1. Is there a difference in the application of direct SLP services through telepractice with respect to an SLP's age, years of working experience, and employment system?
2. Why don't some SLPs offer telepractice to certain clients?
3. Why do some clients not accept telepractice?
4. Does an SLP's age, years of working experience, employment system, and whether or not they conduct online direct therapy influence their satisfaction with telepractice?
5. Do SLPs consider themselves sufficiently competent to conduct telepractice after only one month of experience?

## METHODS

### PARTICIPANTS

A total of 255 speech and language pathologists (SLPs) in Croatia participated in the study. This number represents a quarter of all SLPs in the country. The SLPs came from various regions, belonged to different age groups and worked within different systems (both public and private). The Survey was pre-designed to automatically exclude participants who reported that they had provided no telepractice services (*n=*65). The answers of these 65 SLPs were analysed with respect to the general demographic and employment data (questions 1-7). Other analyses included data provided by the remaining 190 participants who were conducting telepractice during COVID-19. Demographic details of all respondents are presented in Table 1.

**Table 1:** Demographic Characteristics of all Survey Respondents (n=255) and of Those Whose Data Were Finally Analysed as They Completed the Entire Survey (n=190)

All participants (*n=*255)		Participants who implemented telepractice during COVID-19 (*n=*190)			
***Gender***			
Female	249 (97.65%)	Female	188 (98.9%)
Male	6 (2.35%)	Male	2 (1.1%)
***Age groups***			
20-30 yrs.	86 (33.7%)	20-30 yrs.	68 (35.8%)
31-40 yrs.	90 (35.3%)	31-40 yrs.	65 (34.2%)
41-50 yrs.	43 (16.9%)	41-50 yrs.	35 (18.4%)
51-66 yrs.	36 (14.1%)	51-66 yrs.	22 (11.6%)
***Years of working experience***			
0-9 yrs.	127 (49.8%)	0-9 yrs.	100 (52.6%)
10-19 yrs.	74 (29.0%)	10-19 yrs.	50 (26.3%)
20-39 yrs.	54 (21.2%)	20-39 yrs.	40 (21.1%)
***System of employment***			
Health care	96 (37.6%)	Health care	74 (38.9%)
Education	90 (35.3%)	Education	65 (34.2%)
Social	21 (8.2%)	Social	15 (7.9%)
Private practice & NGO	48 (18.8%)	Private practice & NGO	36 (18.9%)

Most participants' caseloads constituted preschool children in the age range from 3-7 years (*n=*120; 63.2%). The other participants worked either with school-aged children (*n=*55; 28.9%), children under the age of 3 years (*n=*8; 4.2%), or with the adult population (*n=*7; 3.7%).

Only 4.2% of the sample reported receiving education in telepractice via webinar or in-person training before the COVID-19 pandemic.

### MATERIALS

The *Survey on the Implementation of Remote Speech and Language Therapy Services (or telepractice) During COVID-19 Related Restrictions*, henceforth referred to as “the Survey,” *was* specifically designed for this study. It was structured to be both user-friendly (i.e., brief and easy to complete) and informative (i.e., to include the topics of interest, namely socio-demographic data and key components of telepractice such as client selection, preparation for telepractice, and selection of technology). The Survey was constructed via the SurveyMonkey platform. The first version was piloted by one SLP. After her valuable comments on the structure and terminology, the Survey was revised. The final version contained 31 questions divided into three sections which corresponded to the study goals: (1) demographic data of the respondent (SLP) and his/her employment system (q. 1-7); (2) data on provision and features of telepractice, as well as on its beneficiaries (q. 8-21); and (3) data on the general opinion and level of satisfaction with this type of service provision (q. 22-31). Most questions were free-choice or yes-no questions, with some Likert-scale and open-ended type questions. In all, 27 questions were mandatory, while the remaining four were optional (i.e., three *if-then* chain-type questions and one comment-type question).

### PROCEDURE

Prior to the study, a request for permission to start data collection was sent to the Board of Directors of Croatian Logopaedic Association [CLA]. After the Ethics Approval (23^rd^ April 2020), a public e-mail invitation was sent to the members mailing list by CLA administrators. The email contained the purpose and the link to the Survey. Participation was optional, with anonymity of personal data and confidentiality guaranteed. A link to the Survey was available from the beginning of May 2020 until the middle of the same month as by that time the first relaxation of restrictive measures related to SLP service delivery was declared by the National Civil Protection Headquarters. During the period of data collection most of the respondents already had approximately one month of telepractice experience. Each respondent completed the Survey individually. Survey completion took approximately 10-15 minutes.

### DATA ANALYSES

Prior to data analyses all individual answers were coded and then transferred to a statistical software package. Using IBM SPSS Statistics version 23.0 (IBM, 2015) several analyses of the collected data were carried out in accordance with the study aims. Analyses included descriptive statistics, chi-square and t-tests comparisons, and simple analysis of variance, as appropriate.

## RESULTS

The first question of the study was to observe whether there is a difference in the application of direct SLP services through telepractice with respect to SLPs' age, years of working experience, and the employment system. The initial step was to extract frequency distributions of answers that relate to the type of services SLPs provided via telepractice. The respondents could mark whether they conduct direct services (i.e., meaning they work directly with the client) or not (e.g., they focus on counselling or on providing indirect services via family members). From the sample of 190 respondents who implemented telepractice during the COVID-19 pandemic, 115 (60.5%) stated that they provide direct services, while 75 (39.5%) reported that they do not work directly with the client. Frequency distributions are outlined with respect to SLPs' age, years of working experience and employment system, all provided in Table 2.

**Table 2:** Frequency Distributions Indicating SLPs Who Implement and Do Not Implement Direct Therapy Services with Respect to Age, Years of Working Experience and System of Employment

*Variables*	*Direct services*		
	*Yes (n)*	*No (n)*	*Total (n)*
***Age groups***			
20-30	44	24	68
31-40	42	23	65
41-50	17	18	35
51-66	12	10	22
***Years of working experience***			
0-9 yrs.	67	33	100
10-19 yrs.	27	23	50
20-39 yrs.	21	19	40
***System of employment***			
Health care	53	21	74
Education	24	41	65
Social	4	11	15
Private practice & NGO	34	2	36
***Total (n)***	***115***	***75***	***190***

To compare frequency distributions and to see whether there were differences with respect to age, years of working experience, and system of employment, a chi-square test was employed. Analyses were based on the reported frequencies (Table 2). Results revealed that there were no differences in the conduct of direct telepractice services with respect to age (χ2=3.375; df=3; p=0.337) and years of working experience (χ2=3.724; df=2; p=0.155). On the other hand, differences were observed when systems of employment were compared (χ2=43.502; df=3; p<0.001). SLPs working in health care systems were most likely to implement direct therapy via telepractice, followed by SLPs working in private practice or in an NGO. As for other systems, Survey responses imply that there were more SLPs who chose to implement other types of services (e.g., indirect or consultative), than those who chose to work directly with the client (see Table 2).

**Figure 1:**
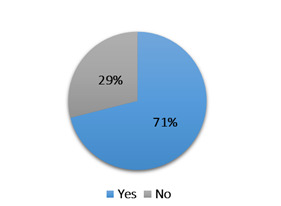
The Proportion of Respondents who Offered Telepractice to all Their Clients during COVID-19 (Yes) and Those Who Did Not (No)

The second question addressed in this study identified reasons behind each professional's decision to not offer telepractice to certain clients. The proportion of SLPs who decided to offer these services to all their clients, and those who did not offer it to all clients, was extracted. These proportions are presented in Figure 1.

**Figure 2:**
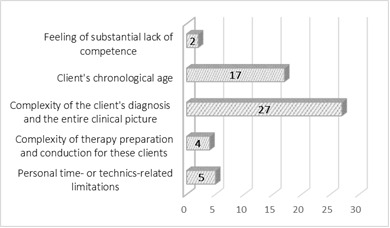
The Frequency (n) of Selected Reasons for Not Offering the Telepractice to Clients

Most respondents offered telepractice to all their clients (71%) because their caseload was relatively homogenous with respect to age and diagnosis. The remaining 29% of the sample deliberately chose to not offer remote services to all clients; their client selection was based on pre-established criteria. While answering the Survey questions respondents were instructed to mark the most relevant factor that influenced their decision not to offer telepractice services to certain clients. The majority of the SLPs singled out complexity of the client's clinical picture and their chronological age as the main reasons; organisational, time- and technology-related factors were chosen to a lesser extent. The distribution of selected answers is presented in Figure 2.

The third question of the study addressed clients who chose not to receive speech and language services through telepractice during the COVID-19. The percentage of client declination of telepractice was relatively high. Up to 61.1% (*n=*116) of the current SLP sample indicated that not all their clients agreed to this sudden and unexpected remodelling of the previous and well-known mode of service provision, whereas 38.9% (*n=*74) of SLPs marked that all of their clients decided to receive services via telepractice during the pandemic. From the SLPs' perspective, the reasons for clients to reject the proposal were few. They seemed mostly related to the lack of technical conditions (*n=*48) and the fact that this service delivery model would require additional engagement of the parents and/or other family members, as well as the clients themselves (*n=*118) who were burdened with the need to restructure their personal and professional lifestyles in less than a month. Interestingly, 12 SLPs marked that their clients decided not to engage in telepractice due to a lack of confidence in its effectiveness.

It appears that some clients decided not to receive telepractice since it required significant engagement of other family members, and not all had the necessary capacities. For this reason, it seemed important to look more closely at the results of the survey question that inspected the client's level of independence during use of telepractice. Findings suggested that only 2.6% of SLPs found their clients to be completely independent participants in telepractice, while 34.8% of SLPs reported that their clients were completely dependent on other family members to provide them with content-related and technical assistance. In addition, 54.7% of SLPs identified their clients as only partially dependent on other persons for content or technical assistance during remote service provision, while 7.9% indicated that their clients were completely dependent on others only for technical assistance, but not for content-related matters.

The fourth question was to investigate professionals' satisfaction with telepractice and specifically, to discern if there was a difference in their level of satisfaction relative to age, years of working experience, employment system, and the fact that they conducted (or not) a direct face-to-face online therapy. To address this question, professionals' satisfaction measured on a Likert-type scale was put in relation to each of these factors, and a series of simple analyses of variance were performed.

**Figure 3:**
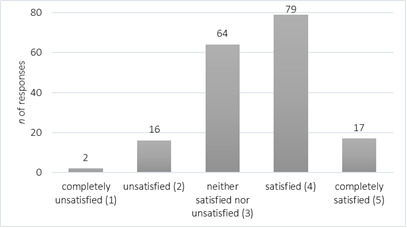
SLPs' Level of Satisfaction with Telepractice in General (n=178)

Of the 190 of the sample who stated that they conducted telepractice during COVID-19 in May 2020, 178 responded to the question inspecting their level of satisfaction. As indicated in Figure 3, most of the participants were either satisfied (4 on a 5-point Likert scale), or their level of satisfaction was moderate (3 on a 5-point Likert scale).

The second step in this research analysis was to conduct a series of one-way ANOVAs, with one factor being the level of satisfaction and the other being different for each analysis: age group (for the first analysis), years of working experience (for the second) and system of employment (for the third). Significant differences were observed only when level of satisfaction was put in comparison relative to the system an SLP was employed in [*F*(3.177)=9.084; p<0.001]. The post-hoc Scheffe test revealed that differences were significant between health care and education system, with SLPs working in health care being more satisfied with telehealth (*M*=3.72; *SD*=0.82 vs *M*=3.13; *SD*=0.74; p<0.001), as well as between education and private practice/NGO, with the latter again indicating a significantly higher level of satisfaction (*M*=3.88; *SD*=0.82 vs *M*=3.13; *SD*=0.74; p<0.001) (see Table 3).

**Table 3:** Level of Satisfaction Distributed Across Systems of Employment

*System*	*N*	*Min*	*Max*	*M*	*SD*
Health care	69	1	5	3.72	0.82
Education	61	1	4	3.13	0.74
Social	15	2	4	3.40	0.63
Private practice & NGO	33	2	5	3.88	0.82

*Note.* 1=completely unsatisfied; 5=completely satisfied

Next, the researchers investigated features of general satisfaction with telepractice, to observe whether there was a difference in the overall level of satisfaction with telepractice between those who decided to implement direct face-to-face online services with their clients and those who used an indirect (counselling) approach. An independent sample *t*-test was conducted. The analysis revealed that difference between these professionals' level of satisfaction was significant [*t*(176)=4.79; p<0.001]; those who worked directly with the client via telepractice were much more satisfied (*M*=3.75; *SD*=0.81) than those who did not (*M*=3.18; *SD*=0.74).

The fifth and final question investigated whether SLPs generally perceived themselves as competent to conduct telepractice after only a month of experience. From the current sample of participants who indicated they conducted telepractice (*n=*190), 73% felt competent enough to do so, while 27% did not. When these two groups (according to self-perceived competence) were compared also with respect to their above-mentioned satisfaction with telepractice, the differences were significant [*t*(176)=5.243; p<0.001)]. SLPs who felt competent were generally more satisfied with telepractice (*M*=3.71; *SD*=0.75) than those who did not feel competent (their level of satisfaction was lower; *M*=3.02; *SD*=0.84).

Despite that very few respondents (only 4.2%) received additional education in telepractice before the COVID-19 pandemic (see Participants section), they generally recognized its importance. More specifically, 68.5% of the sample considered additional education in telepractice absolutely necessary, and 6.7% reported that education is needed only if one wishes to implement it in work with certain types of disorders (e.g., childhood apraxia of speech (CAS), ASD or with generally more complex clinical pictures or comorbid conditions). On the other hand, 24.7% found additional training for the conduction of remote therapy unnecessary.

## DISCUSSION

The COVID-19 pandemic brought new working conditions to which speech and language pathologists had to adapt quickly and efficiently, and telepractice was the only option for providing speech and language services during quarantine. The aim of the current study was therefore to gain an insight into how SLPs in Croatia coped with the implementation of telepractice during the COVID-19 crisis, in the phase of a sudden and complete lock-down.

The results showed that 60% of SLPs continued to provide direct therapy, and did so via telepractice. Interestingly, age and years of working experience did not appear to be significantly related to this decision, but the system of employment did. SLPs working in health care systems and in private practice were the ones who implemented direct therapy the most. Reasons for this can be found in their job descriptions. SLPs employed in health care and private systems in Croatia primarily conduct so-called prototypical speech and language activities - assessment, diagnostics, therapy and counselling - whereas SLPs working in education have a broader scope of work, which at times exceeds their prototypical professional roles. Namely, the educational system treats SLPs as members of the school team responsible not only for provision of speech and language services, but for promoting examples of good inclusive practice supporting students, teachers and parents, or ensuring adequate social environments for children with speech, language and communication needs. It appears that individuals employed within this system were, due to their usual workload, more inclined to provide general counselling related to education or policies, and to consult and monitor parents or other family members.

The study also aimed to understand SLPs' reasons for not implementing telepractice with certain individuals. This question partially contributes to the understanding of key components of telepractice, such as characteristics of the environment and client selection, i.e. the appropriate choice of individuals who will surely benefit from telepractice (Cason & Cohn, 2014). There are many limitations - physical, cognitive, sensory and communication - which may affect a client's ability to participate in speech and language services via telepractice. According to the responses provided by SLPs in the survey, precisely these factors stood out as main reasons for choosing not to provide services to some clients via telepractice.

The percentage of clients that declined telepractice was relatively high. Only 38.9% of SLPs reported that all their clients decided to receive telepractice services during the lock-down. The main reasons for rejection were the lack of technical conditions and the need for additional engagement of parents and/or other family members. These results corroborate findings reported by Plantak Vukovac (2016) who singled out technical conditions and additional engagement of family members as the main limitations of telepractice. These results are particularly understandable when observed in the context of the COVID-19 pandemic. During lock-down the entire family was obliged to work and attend school lessons from the same place, with news about the pandemic being reported to them on a daily basis. Digital literacy is another important consideration. According to Eurostat, 82% of the Croatian population has internet access (Croatian Bureau of Statistics [CBS], 2018) but only 57% of the population aged 10 to 80 years is digitally literate. With increase of chronological age, the number of ICT and internet users decreases, and after the age of 65 this percentage drops below 10%. Our data indicates that certain clients could not be included in remote services due to the lack of digital competence or due to the client's dependence on other family members who possess this competence in order to receive services through telepractice. A small number of respondents indicated that certain clients rejected telepractice because they did not believe it to be effective. It is still a common belief in Croatia that direct in-person face-to-face individual therapy is the most effective, while other approaches, e.g., group or indirect, are less favourable. Nevertheless, parents can change their perspectives, biases, and attitudes when they are included in the child's progress, as stated in a recent pre-experimental study on group-based and indirect therapy provided to children with developmental language disorder (DLD) (Matić et al., 2018). This implies that it is necessary to continuously work on promoting various models of speech and language therapy, especially those for which effectiveness has been confirmed (Law et al., 2017; Law et al., 2019).

Observing the broad motives for not offering and not agreeing to receive telepractice, it seems that both were associated with challenges related to *setting* (e.g., time, place, technical conditions) and *client selection* (e.g., age, complex clinical picture), and much less to the lack of certainty in effectiveness or confidence in true potentials of telepractice.

Findings on SLPs' satisfaction suggest that the level of satisfaction was relatively diverse. This seemed to be dependent on the system a person worked in, and whether or not the professional provided direct therapy to the client. Both these notions can be linked to the first finding of the study. SLPs employed in health care and private practice are more satisfied with telepractice than those working in the education system, partially due to the fact that telepractice is a much more convenient tool for the application of direct therapy (provided to a greater extent by SLPs in health care and private practice), while it is much less convenient for SLPs in education. As highlighted earlier, aside from providing speech and language services to the clients, SLPs in education systems implement interventions that support students in meeting the demands of the curriculum (Powell, 2018). Therefore, these professionals engage with more people and their daily working routine encompasses diverse professional responsibilities. Moreover, satisfaction is linked to the concept of self-competence. Professionals who felt competent to provide telepractice also reported higher levels of satisfaction. After only one month of work under completely new and extreme conditions, SLPs did adjust and started gaining a certain level of telepractice competence. Consequently, their level of satisfaction with telepractice also increased. Those professionals who reported a lack of competence also felt less satisfied with telepractice.

The practice landscape has rapidly changed and the usual way SLPs, as professionals, organize their work and engage socially with their clients has been seriously challenged as a result of the COVID-19 pandemic. Job descriptions and the entire scope of professional obligations will most likely experience substantial change, as well. The education system will need to follow these tendencies, which is something that SLPs, at least those included in the current study, became aware of very recently. Almost 70% (including those who conduct direct online work and those who provide counselling to advise family members) feel that competency with telepractice requires additional training.

## LIMITATIONS AND FUTURE DIRECTIONS

The current study presents insights into telepractice in Croatia during the COVID-19 pandemic. Since it offers a rather brief overview of practice during this period, certain limitations are evident. First, the Survey was designed specifically for SLPs, so there is no data on client perception. Even questions that inspect reasons for clients' refusal of telepractice were answered by the SLPs. It would be worthwhile to observe in more detail the exact client-related factors that contribute to acceptance (or refusal), as well as the successful application of telepractice with respect to socio-economic and other individual and familial characteristics of the client. Also, analysis related to other aspects (e.g., the application of technologies) was not included in the analysis. Rather, the focus was mainly on client selection and preparation for telepractice.

Future studies should investigate other concepts, including ethical and environmental aspects related to the use of telepractice for delivery of speech and language services. This is indeed very important, especially if implementation of telepractice continues to increase as a result of recent events. Since countries worldwide still significantly differ in educational and legislative aspects regarding provision of telepractice, it would be interesting to observe changes in each of these areas in the months and years to come.

## CONCLUSION

Until recently, telepractice has been completely understudied in Croatian SLP settings. However, in the recent months it has received more attention due to its applicability in response to the COVID-19 pandemic. Times of crises do not allow for the usual approach to services and therefore all SLP services require additional adjustment.

This study is the first to investigate telepractice in Croatia, and the results must be interpreted in the context of the extreme conditions in which the data was gathered. Nevertheless, this was the exact goal: to examine SLPs' reactions to the COVID-19 pandemic and to explore their perceptions, insights, and preparation for telepractice. This first step in the research process provides a foundation for forthcoming studies and clinical work, which will likely include increasing use of telepractice in the future.

The results suggest that SLPs in Croatia, despite their relative lack of additional education and experience in telepractice, do approach it thoroughly. They contemplate and ponder over client selection, which they base largely on age and complexity of clinical picture, and they question and reflect on their own competence. Other factors that shape preparation for telepractice are relatively out of SLPs' control (e.g., their system of employment); this may indirectly contribute to feelings of satisfaction and competence, or the lack thereof. Benefits of telepractice most often reported in studies are equity of services, mitigation of distance and travel issues, and transfer to natural settings. Nonetheless, key components of telepractice need to continuously be investigated in-depth, as all services, whether provided in-person or remotely, must rely upon a strong evidence base. Education and legislation should follow the prevailing trends, and ideally be one step ahead.
